# Effects of essential oil (blended and single essential oils) on anti-biofilm formation of *Salmonella* and *Escherichia coli*

**DOI:** 10.1186/s40781-017-0127-7

**Published:** 2017-02-19

**Authors:** S. Y. Oh, W. Yun, J. H. Lee, C. H. Lee, W. K. Kwak, J. H. Cho

**Affiliations:** Division of Food and Animal Sciences, Chungbuk National University, Cheongju, Chungbuk, 361-763 South Korea

**Keywords:** Biofilm, Essential oil, *Escherichia coli (E. coli)*, *Salmonella*, Pigs

## Abstract

**Background:**

Biofilms were the third-dimensional structure in the solid surface of bacteria. Bacterial biofilms were difficult to control by host defenses and antibiotic therapies. *Escherichia coli (E. coli)* and *Salmonella* were popular pathogenic bacteria that live in human and animal intestines. Essential oils are aromatic oily liquids from plant materials and well known for their antibacterial activities.

**Method:**

This study was conducted to determine effect of essential oil on anti-biological biofilm formation of *E. coli* and *Salmonella* strains in in vitro experiment. Two kinds of bacterial strains were separated from 0.2 g pig feces. Bacterial strains were distributed in 24 plates per treatment and each plates as a replication. The sample was coated with a Bacterial biofilm formation was.

**Result:**

Photographic result, *Escherichia coli (E. coli)* and *Salmonella* bacteria colony surface were thick smooth surface in control. However, colony surface in blended and single essential oil treatment has shown crack surface layer compared with colony surfaces in control.

**Conclusion:**

In conclusion, this study could confirm that essential oils have some interesting effect on anti-biofilm formation of *E. coli* and *Salmonella* strains from pig feces.

## Background

Biofilms was defined as communities of microorganisms that grow soaked up in a matrix of third-dimensional structure in solid surface on film, adhered to inert material or live tissue. These modes could consist of single or different bacterial species and even different genera. Bacteria biofilms are known to be developed in host epithelial cell, bone, tooth, walls of a blood vessel and medical appliance [1]. Bacterial biofilms are difficult to detect in routine diagnostics and are inherently tolerant to host defenses and antibiotic therapies. In addition, biofilms are reported to be a possible means to increase the resistance against antibiotic by promoting horizontal gene transfers [2]. These biofilms might contain spoilage and pathogenic microorganisms [3].

*Escherichia coli (E. coli)* and *Salmonella* are popular pathogenic bacteria. *E. coli* are bacteria that live in human and animal intestines. *E. coli* produce a toxin called shiga toxin, which causes illness in humans. The symptoms of *E. coli* infection include the sudden onset of cramps and abdominal pain, followed by diarrhea. Accoding to Mead and Griffin [4]), *E.coli* infections are detected various disease symptoms of human and animals. Moreover, *Salmonella* are reported to be the second most common important bacterial cause for intestinal infection in the United States. *Salmonella* infection usually occurs when a person eats food contaminated with the feces of animals or humans carrying the bacteria. Non-typhoidal *Salmonella* are the most common form, and it is carry by both humans and animals. Santos et al. [5] reported that typhoid fever and enteritis of *Salmonella* symptom could infect to human and animal easily. The most common symptoms include diarrhea, abdominal cramps, and fever.

Two strategies to get rid of biofilm include chemical and biological methods. In chemical method, biocides and disinfectants have been reported to be the principal weapons to control unwanted biofilms [6]. In biological method, use the selected natural products which originate in form plants (e.g. essential oil). It has been suggested that essential oils originating from plants consist of, mixtures of numerous organic chemicals, that has the ability to inhibit growth [7]. Moreover, essential oils were aromatic oily liquids from plant materials and well known for their antibacterial activities. Essential oils existed promising natural ingredients for the food industry due to their preservative and antimicrobial effects [8]. In a study by Vázquez-Sánchez et al. [9], essential oil is renowned for preventing the production of *Staphylococcus aureus* biofilms and another studies reported that essential oil, has been reported to inhibit *Pseudomonas aeruginosa* biofilm formation [10]. Therefore, the objective of the study was to determine the effects of using single or blended essential oil to decompose biofilm layer of *E.coli* and *Salmonella* *in vitro*.

## Methods

### Essential oil extract

The blended essential oil used in the present experiment was in powdered form and obtained from commercial company (Aromex-me; Yuhan Corporation, korea 2015). In brief, essential oil powder was mixed in DW, 1:1 ratio to make a solution. The powdered essential oil solution in water was centrifuged at 3000xg for 20 min, and the oil layer was pipetted. Single essential oils tested in this experiment were (90% of pure oregano, thymol, cavacrol).

### Bacterial strain

*Salmonella* sps and *E.coli* sps were isolated from pig feces by diluting 0.2 g of feces in 1 ml of distilled water and then 200ul of diluted solution was smeared in petridish having *Salmonella Shigella* agar and MacConkey agar respectively. These agar plates were incubated at 37 °C, for 24 h for *Salmonella* sps isolation and 12 h of *E.coli* sps isolation. The culture of bacteria was made from Sterile laboratory of Chungbuk National University.

### Biofilm formation and experimentation

For the formation of biofilm form the *Salmonella* sps and *E.coli* sps the colonies formed in the agar plates were incubated at 37 °C for 7 days. After 7 days of incubation, the expansion of bacteria out layer was observed.

Scanning electron microscopy (SEM) was used to investigate the structural modifications of biofilms after treatment with EOs. For biofilm formation, 5 ml of overnight *Salmonella* sps and *E.coli* sps were added to 6-well macrotitre plates. Sterile coverslips were placed in the wells and served as the attaching surface for the cells. The plates were incubated for 4 h at 30 °C, then the supernatant was removed, and plates were rinsed with physiological saline. For treatment of *Salmonella* sps and *E.coli* sps biofilms, 200ul of blended or single essential oil solution of MIC/2 concentration was added, Control samples contained only liquid culture media. The macrotitre plates were incubated for 24 and 48 h. After incubation, the supernatant was removed, and the wells were washed with physiological saline. The preparation of the samples for electron microscopy was performed as follows: soaking of the sample with 2.5% glutaraldehyde in 0.05 mol/L cacodylate buffer (pH = 7.5), for 2 h at room temperature, followed by dehydration using different ethanol concentrations: 50, 70, 80, 90, 95 and 98%. Each ethanol treatment lasted for 2 × 15 min at room temperature. The next step was dehydration with t-butyl–100% ethanol solution in 1: 2, 1: 1 and 2: 1 ratios. For each ratio, the dehydration lasted for 1 h at room temperature, then dehydrated with absolute t-butyl alcohol for 2 × 1 h at room temperature; and changed to new t-butyl. The sample was stored at 4 °C for 1 h and freeze–dried overnight.

## Results and discussion

The anti-biofilm effect of essential oil was observed in this study based on the observation made using SEM.

**Fig. 1:**
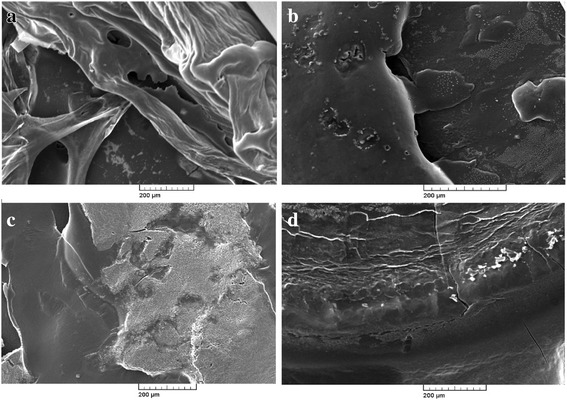
E.coli and Salmonella biofilm control & blended essential oil treatment (**a**) E.coli biofilm control (**b**) E.coli blended essential oil treatment (**c**) Salmonella biofilm control (**d**) Salmonella blended essential oil treatment

**Fig. 2:**
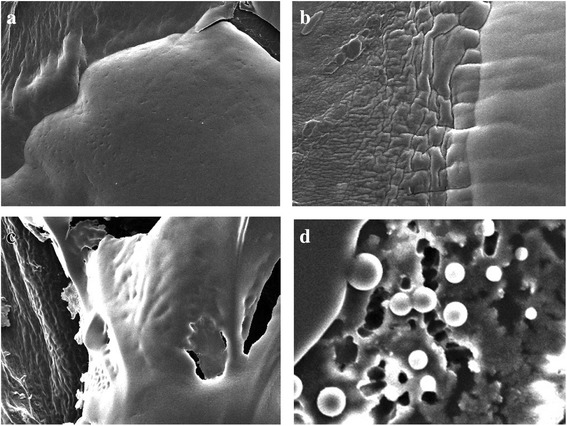
E.coli biofilm control & single essential oil treatment (cavacrol, oregano, thymol) (**a**) E.coli biofilm control (**b**) E.coli cavacrol treatment (**c**) E.coli tymol treatment (**d**) E.coli oregano treatment

**Fig. 3:**
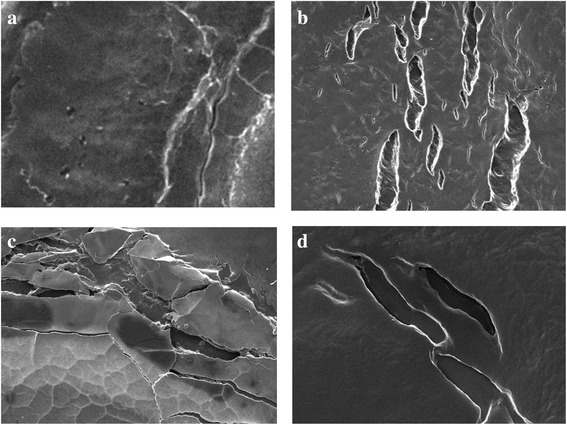
Salmonella biofilm control & single essential oil treatment (cavacrol, oregano, thymol) (**a**) Salmonella biofilm control (**b**) Salmonella cavacrol treatment (**c**) Salmonella tymol treatment (**d**) Salmonella oregano treatment

Photographic result shows the, biofilm formation and experimentation in *E.coli* and *Salmonella*. In Fig. 1 (a) and (b) pictures depicts *E.coli* surface, (a) shows thick ruggedly surface, but EO treatment (b) shows thin and smooth surface. The same result in *Salmonella*, (c) and (d) pictures, (d) is smooth surface rather than (c). Results shows that, essential oil affect in removal biofilm. Figures 2 and 3 show, in sight surface crack in colony. This study shows that essential oil has an effect on biofilm because blend essential oil and single essential oil show anti-biofilm activity.

Results of our study showed, inhibition of biofilm when essential oil used in *E.coli* and *Salmonella*. Compared with pictures, Figs. 2, 3 are better than Fig. 1 in anti-biofilm formation (In Figs. 2 and 3 picture have deep crack). Also, this photographic result has found that using single E.O is better than using blend E.O.

In recent years, there is an increase trend of interest in the strategies to treat biofilm and essential oil has been considered effective for substitution of antibiotics. Essential oils are volatile compounds with antimicrobial properties constituting non-supportive media for the growth of many bacteria and fungi [11]. Medicinal plants are considered as potential sources of new chemotherapeutic drugs because of their diverse and non- toxic effect [12].

In this study, we observed suppression of biofilm through essential oils. Carvacrol and thymol are principal components of oregano and thyme oils. They have a very similar chemical structure consisting of a system of hydroxyl group on the phenolic ring, which are required to elicit strong antimicrobial activity [13]. A variety of researches have, shown that the biofilm could be removed effectively by essential oils for example, cinnamon oil [14, 15], thymol [16], eucalyptus ([17, 18]; Mathur et al.., 2014), tea tree ([19];). We have used essential oil as powder form of feed additives (Aromex-me) and three kinds of essential oils (oregano, thymol, cavacrol). In this study, feed additive was composed of phytogenic active substances – like essential oils, herbs and spices and 100% phytogenic solution that could use as an antibiotic alternative in swine. Oral et al. [20] reported that carvacrol and thymol which are the principal phenolic components of oregano oil might have played a role in the inhibition of biofilm formation by micro bacteria.

## Conclusion

In conclusion, this study could be used as a step forward to confirm that essential oils have a positive effect on anti-biofilm formation of *E. coli* and *Salmonella*. Moreover, single essential oil has a better result on anti-biofilm formation than blended essential oil, Thymol and Oregano essential oil has a better result on anti-biofilm formation of *E. coli* than Cavacrol essential oil; however, Cavacrol and Thymol essential oil has a better result on anti-biofilm formation of *Salmonella* than Oregano essential oil. But the findings of this study is judged through one SEM photo which is the limitation of this study. Thus, more researches are needed to confirm the effects of essential oil in microbial biofilm formation *in-vitro* in swine.
